# Primary Gastrointestinal Stromal Tumor Presenting as an Isolated Lung Mass

**DOI:** 10.7759/cureus.8343

**Published:** 2020-05-28

**Authors:** Allison Mootz, Thuy Nguyen, Keshav Poddar, Anuj Goel

**Affiliations:** 1 Anesthesiology, University of Texas Southwestern Medical Center, Dallas, USA; 2 Internal Medicine, Texas College of Osteopathic Medicine, Fort Worth, USA; 3 Internal Medicine, Methodist Health System, Dallas, USA

**Keywords:** gastrointestinal stromal tumors, lung mass, extraintestinal gastrointestinal stromal tumors, malignancy, tyrosine kinase inhibitors, immunohistochemical stain

## Abstract

Gastrointestinal stromal tumors (GISTs) are rare gastrointestinal (GI) tumors, representing a small portion of soft tissue tumors of the abdominal cavity. Extraintestinal gastrointestinal stromal tumors (EGISTs) are uncommon forms of GISTs that present outside the GI tract. There have only been a rare number of reported cases of EGIST presenting above the diaphragm. We present the case of a 50-year-old female with shortness of breath, and found to have bilateral pleural effusions and left-sided lung mass. The initial lung mass aspiration was negative for malignancy; yet, pleural fluid was suggestive of malignancy, and repeat biopsy and immunohistochemical stain were diagnostic for GIST. Ultimately, the patient underwent video-assisted thoracoscopic surgery, pleurodesis with doxycycline, and adjuvant therapy with imatinib. This is a report of primary EGIST presenting as an isolated lung lesion with no involvement of the GI tract. In patients with suspected malignancy, it is of paramount importance to obtain a detailed history, including remote signs and symptoms, while performing a thorough work-up. Especially in the lung where initial biopsies can be skewed due to inflammation and atelectasis, repeat biopsies may be necessary to obtain an accurate diagnosis.

## Introduction

Gastrointestinal stromal tumors (GISTs) are considered a distinct subgroup of tumors within the realm of soft tissue sarcomas [1]. They were once thought to be a form of leiomyomas, leiomyosarcomas, or leiomyoblastoma [2]. It was discovered in 1998 that these tumors originate from the intestinal cells of Cajal in the myenteric plexus, which controls peristalsis [3]. GISTs are now recognized as a distinct entity within soft tissue sarcomas, accounting for only 4%-7% of abdominal cavity soft tissue tumors [1]. They have variable malignant potential, ranging from small, benign tumors to large sarcomas [2]. GISTs most commonly present in the stomach (50%-60%), small intestine (30%-35%), colon and rectum (5%), and esophagus (<1%) [2].

In 1999, Miettinen et al. first described the presence of extraintestinal gastrointestinal stromal tumors (EGISTs), which account for 10% of all GISTs [4]. EGISTs are GISTs that primarily arise outside of the GI tract, with documented cases originating from the prostate, pancreas, kidney, and pericardium. Because GISTs account for only 1% of all GI tumors, EGISTs from metastasis are rare and primary EGISTs are exceptionally uncommon.

The worldwide annual incidence of GISTs amounts to 10 to 22 cases per million people, men and women equally affected, and with a median age between 60 and 65 years. Clinically, one should suspect GISTs in older patients with abdominal pain, bloody stools, nausea or vomiting, constipation or diarrhea, fatigue, dyspepsia, and/or palpable mass [2]. Initial evaluation should include CT with contrast, and as much as 88% of GISTs will stain for both Kit (CD117) and DOG-1 with histopathology and immunochemistry [3]. Laparoscopic resection is preferred and biopsy is standard before initiating neoadjuvant therapy, such imatinib, sunitinib, or ponatinib. Imatinib is a popular choice for neoadjuvant therapy and targets the KIT and PDGF tyrosine kinase activity and indicated for completely resected primary GISTs [5]. It binds to the ATP-binding sites on CD117 and PDGFRA, blocking signal transduction [3].

We present a case of primary EGIST that originates in the pleura, with no involvement of the GI system. To our knowledge, this is the second case of EGIST of the pleura in North America and fourth case of EGIST of the pleura reported internationally.

## Case presentation

A 50-year-old female patient with a past medical history of diabetes mellitus and hypercholesterolemia presented to Hospital A’s emergency room with the chief complaints of shortness of breath and hypoxia. She stated her shortness of breath had been worsening over the past month, most significantly over the past few days. It occurred with exertion and rest. She endorsed a non-productive cough over the same time period. In addition, one year ago she had an episode of melena. Three months ago, she had one episode of hematochezia. She had 30 pounds of weight loss over the past three months. Her last colonoscopy was five to six years ago and she was unaware of the results. She denied fever, chills, hemoptysis, hematemesis, dysphagia, abdominal pain, nausea, vomiting, diarrhea, constipation, chest pain, or travel outside the country. 

The patient’s family history was unremarkable, and her surgical history was pertinent for cholecystectomy, laparoscopic appendectomy, and incisional hernia repair. She smoked three cigarettes per day for seven years and quit five years ago. She denied illicit drug use or alcohol consumption.

**Figure 1:**
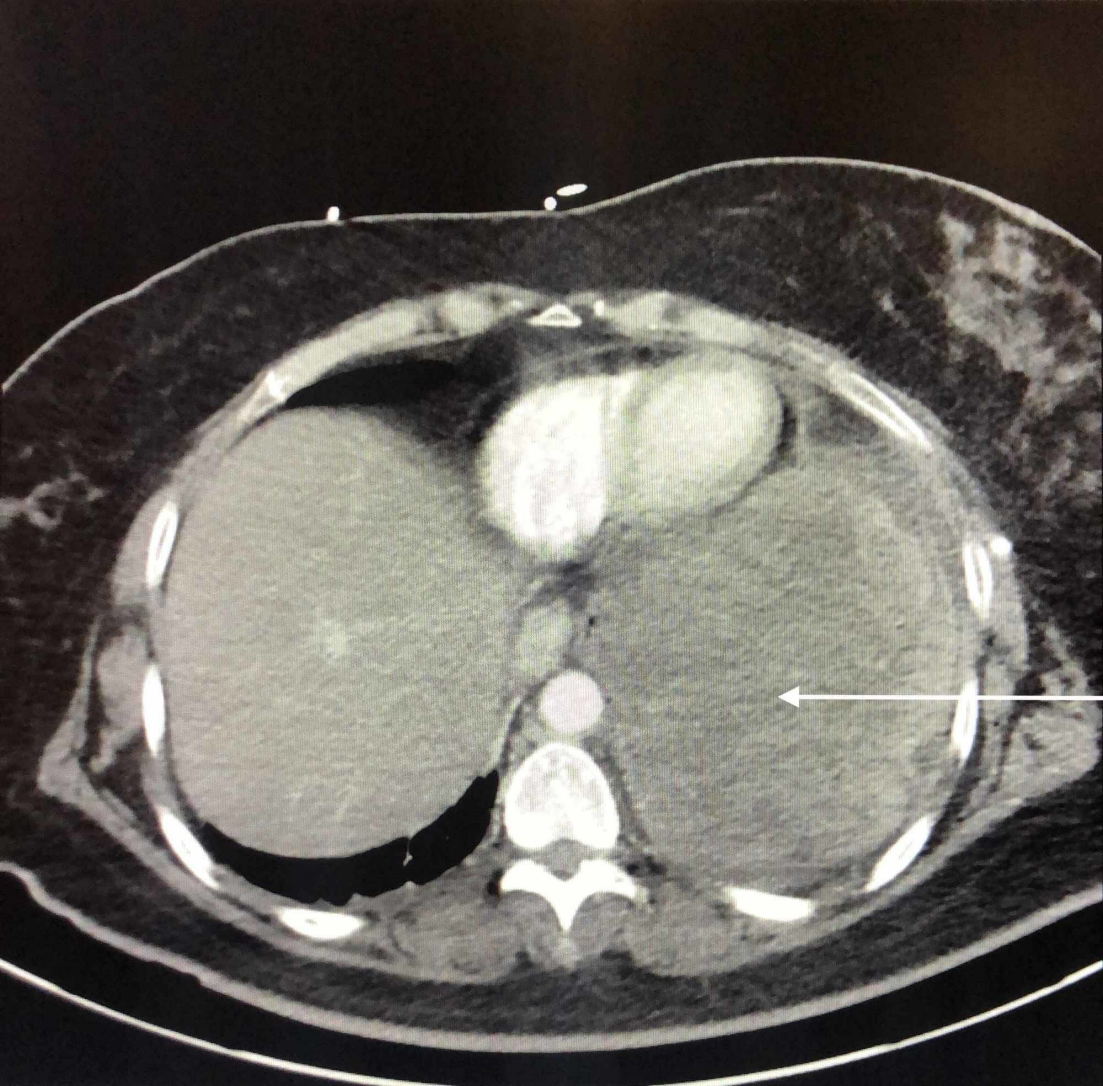
Chest CT shows a large heterogeneous left lower lobe mass with significant attenuation of the medial left lower lobe airway. Findings are concerning for a neoplastic etiology. Additional multifocal ground-glass/nodular opacities throughout the left hemithorax could be secondary to multifocal infection or patchy areas of pulmonary edema. No suspicious right hemithorax nodules are appreciated.

**Figure 2:**
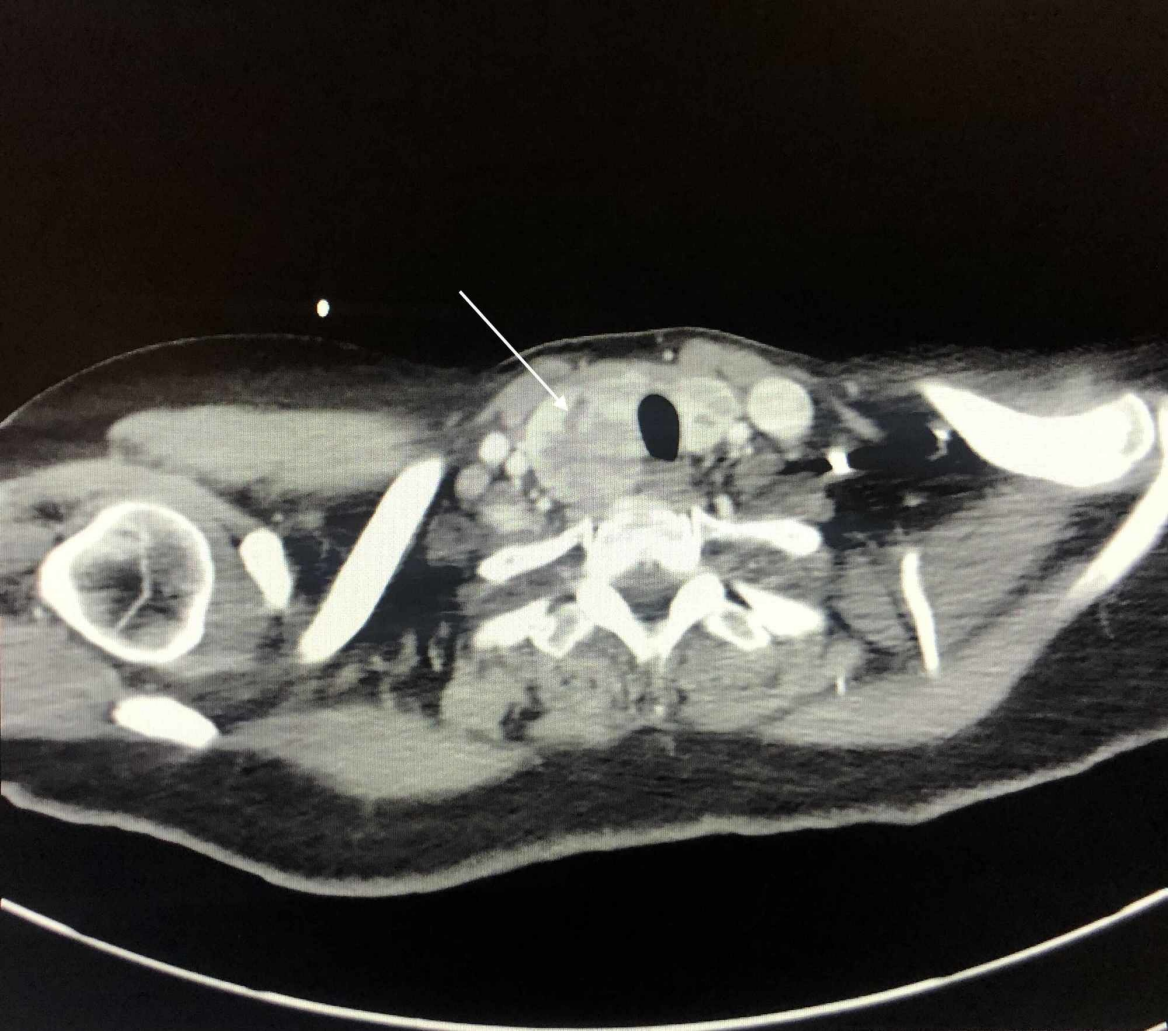
Chest CT shows right thyroid nodule.

Of note, one month ago she presented to an outside hospital with the same chief complaint. At this time, a chest x-ray showed large pleural effusion and possible infiltrate. A chest CT scan also showed a mass of the left lower lobe. A chest tube was placed with 2.5 L of fluid drained. There were no records of the outside hospital sending the pleural fluid for evaluation of transudative versus exudative effusions. She was then transferred to another hospital for higher level of care, further work-up, and chest tube management by specialty services. A repeat chest CT scan with contrast showed a large left pleural effusion, multifocal ground-glass nodular opacities throughout the left hemithorax, and a 12.6-cm heterogeneous left lower lobe mass with significant attenuation of the medical left lower lobe airway, concerning for neoplastic etiology (Figure 1). In addition, it showed a right thyroid nodule (Figure 2).

**Figure 3:**
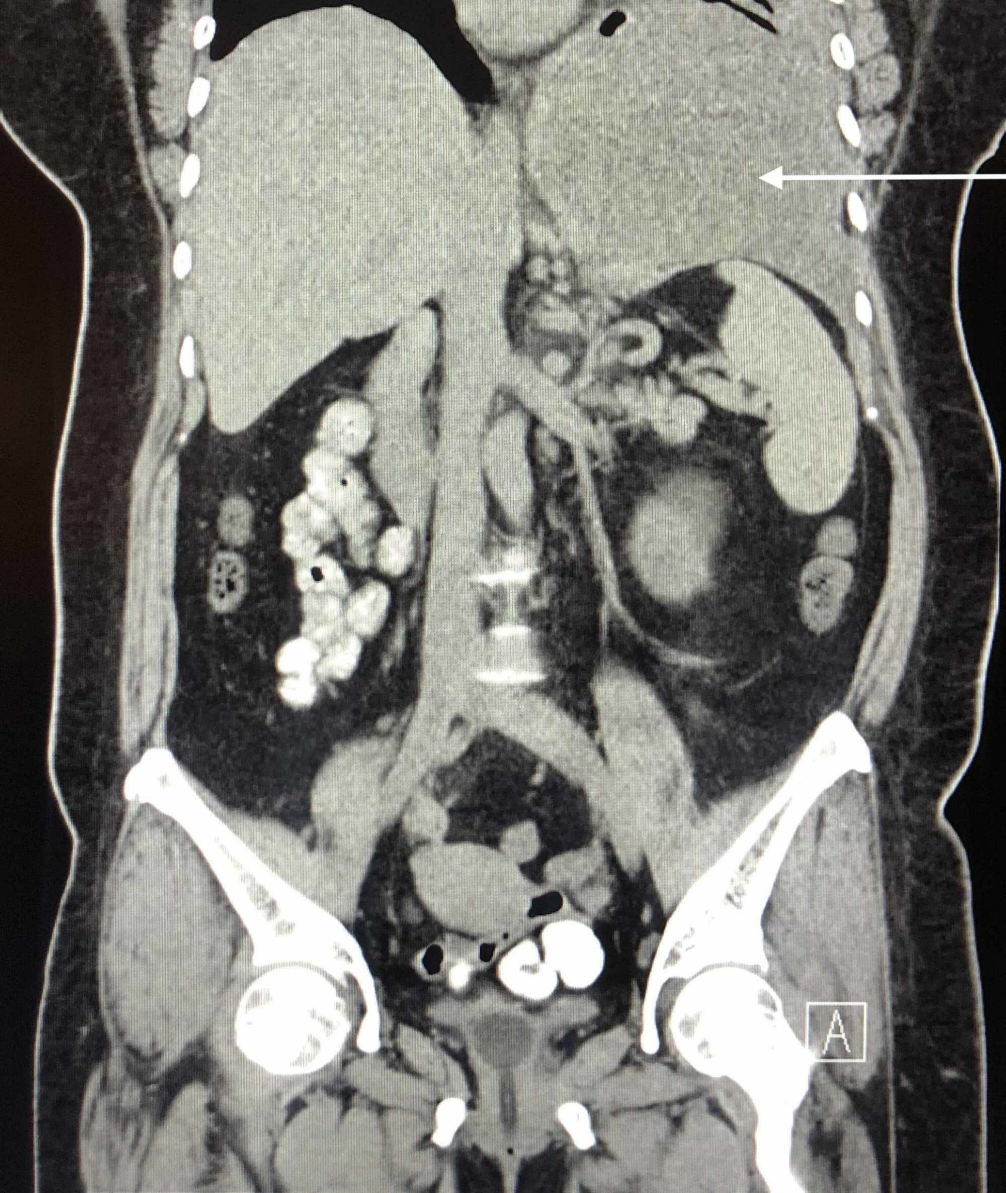
Abdomen and pelvis CT shows left lung mass isolated superior to the diaphragm.

A CT of the abdomen and pelvis showed no acute findings, suspicious lesions, or masses in the abdominal cavity; lung mass was appreciated superior to the diaphragm (Figure 3). A fine needle aspiration of the lung mass was performed revealing no evidence of malignancy. The chest tube was removed four days later. Her hypoxia resolved and she was discharged. 

**Figure 4:**
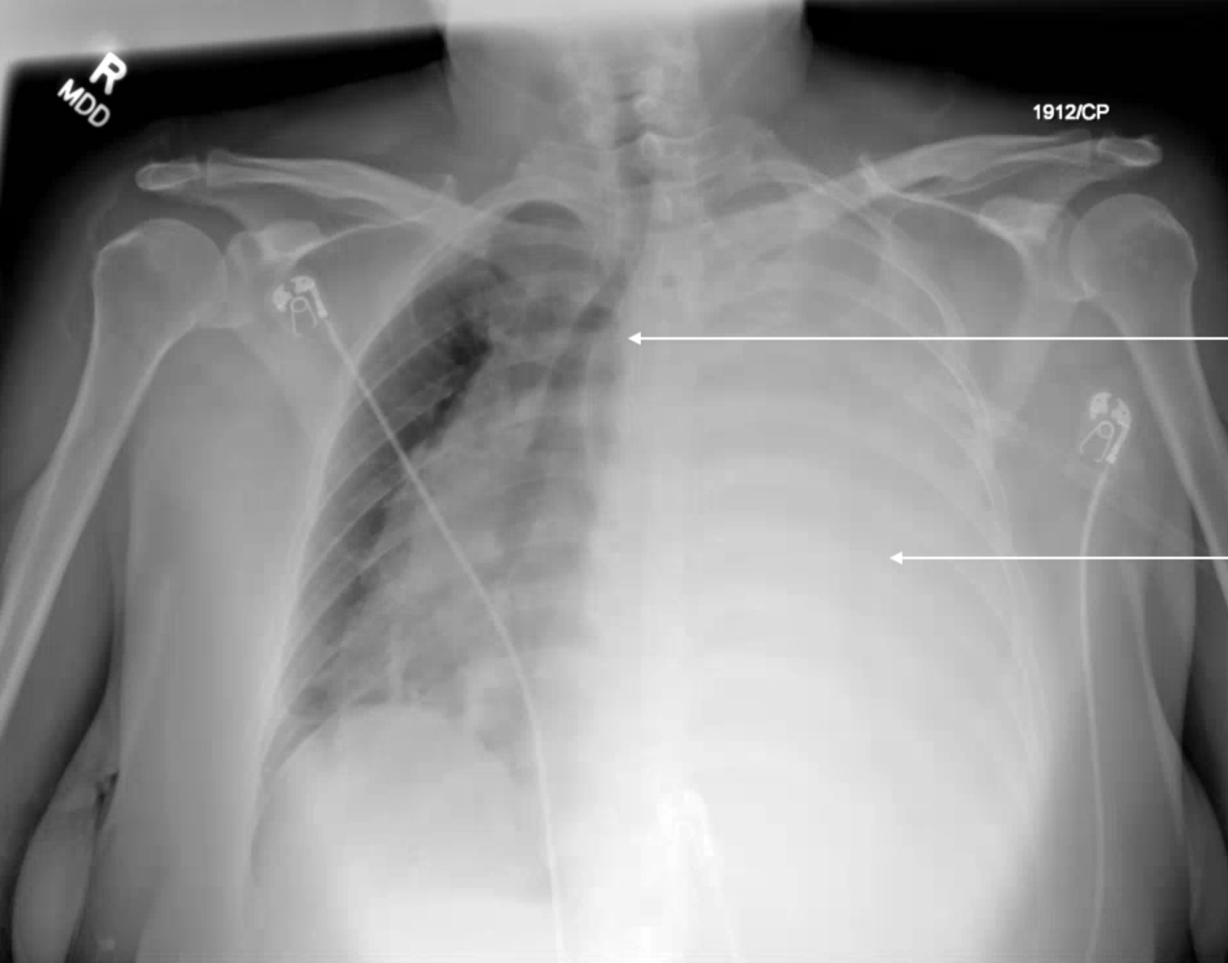
Chest x-ray showing complete left hemithoracic opacification with mass effect including rightward mediastinal shift.

Upon presentation at Hospital A, her vital signs showed she was afebrile, tachycardic, tachypneic, and saturating at 98% on 2 L of nasal cannula, with no oxygen requirement at baseline. Pulmonary exam revealed decreased breath sounds on the entire left side compared to right. No audible rales or rhonchi were auscultated. Her complete blood count (CBC), comprehensive metabolic panel (CMP), lactic acid, and procalcitonin were all within normal limits. A chest x-ray showed an interval near complete left hemithoracic opacification with mass effect including rightward mediastinal shift (Figure 4). She was admitted for her left-sided pleural effusion and work-up of her lung mass, which was concerning for malignancy.

**Figure 5:**
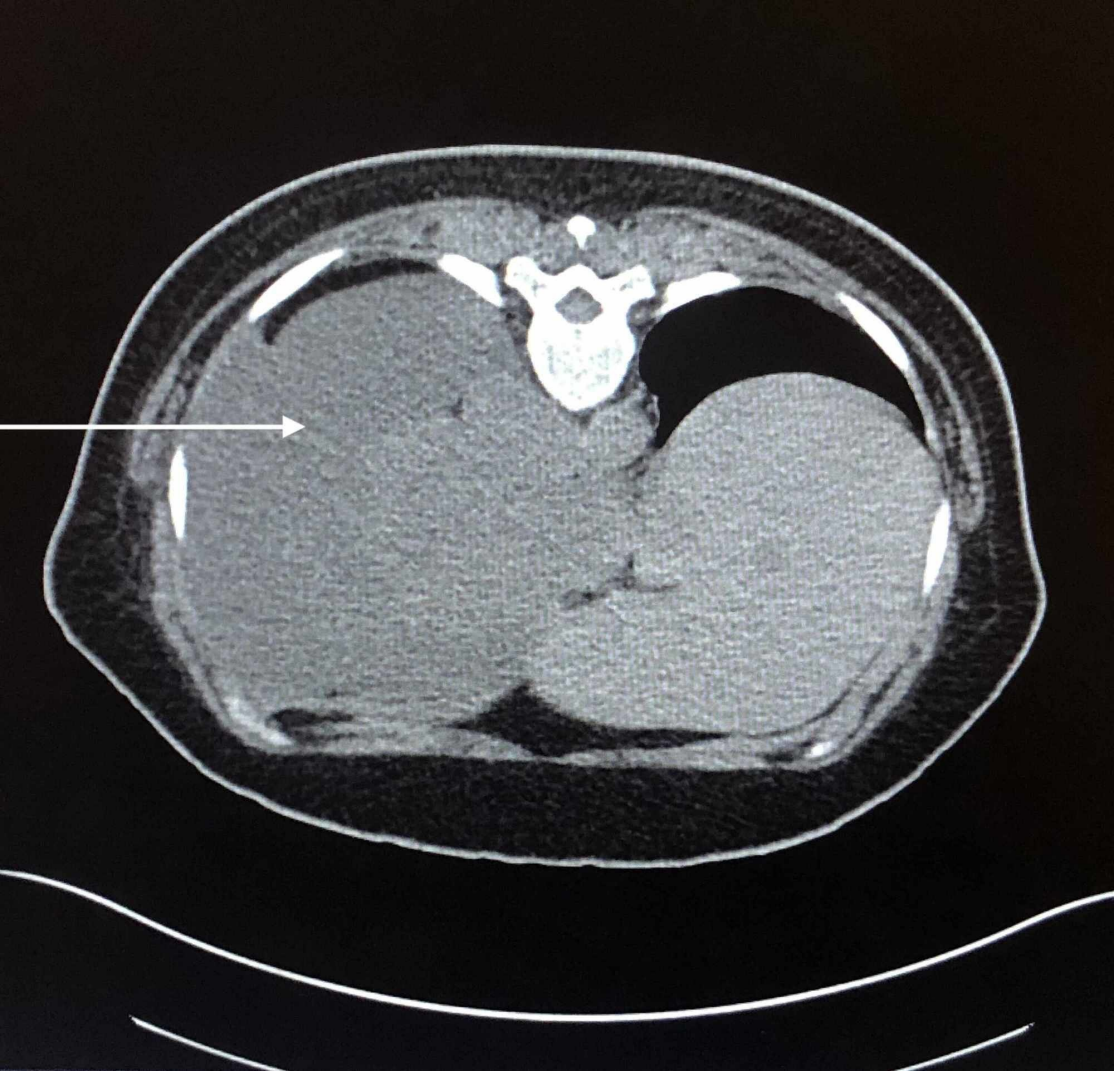
CT-guided thoracentesis and biopsy imaging shows left lung mass.

**Figure 6:**
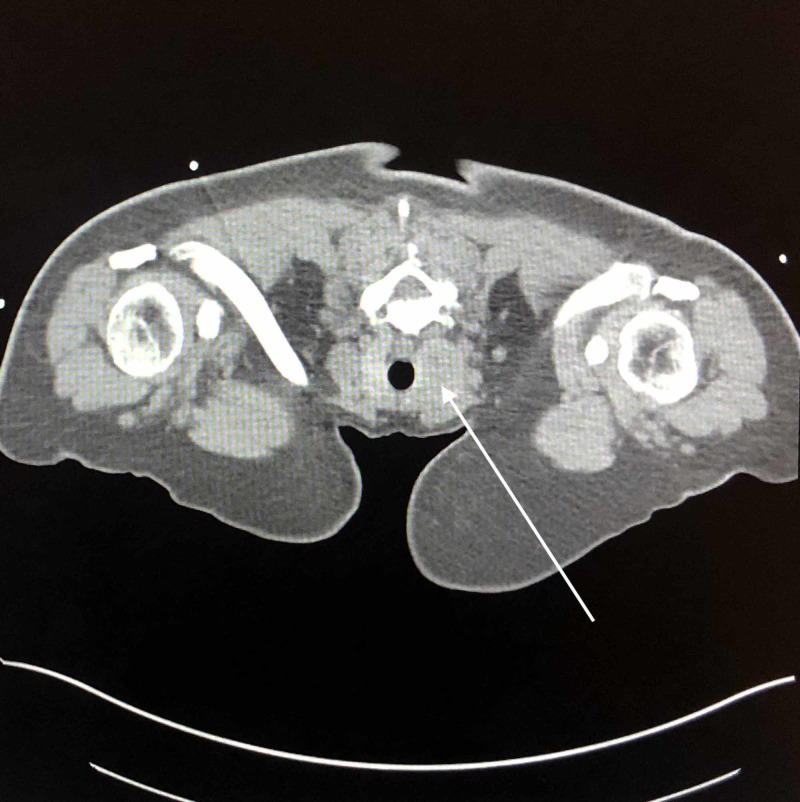
CT-guided thoracentesis and biopsy imaging for lung mass shows right thyroid nodule.

A CT-guided lung biopsy for re-evaluation of the left lower lobe mass was performed. Imaging showed the left lung mass and right thyroid nodule (Figures 5, 6). Also, a left thoracentesis was performed with 2 L of bloody fluid removed. On hospital day 3, it was found that her insurance was not accepted by Hospital A and was transferred to Hospital B. 

At Hospital B, a repeat CT of the chest again showed the massive left pleural effusion, the large left heterogeneous enhancing mass in the lower hemithorax within the pleural space, right mediastinal shift present, and the right thyroid nodule. No thyroid ultrasound was performed at this time. A CT of the abdomen and pelvis was normal. Only radiology reports were available. Hospital B's imaging results were similar to Hospital A's imaging results, as they were performed three days apart. 

Interventional radiology performed a left-sided lung core needle biopsy, which showed tumor morphology and immunohistochemical staining pattern compatible with epithelioid type GIST, grade II, high grade. The immunohistochemical stains were positive for CD117, DOG-1, and CD34, and weakly positive for SMA and CAM5.2. The initial thoracentesis pleural fluid showed mixed inflammation, including eosinophils, rare reactive mesothelial cells, and negative for malignancy. However, repeat thoracentesis drained 1.7 L of sanguineous fluid from her left chest and the pleural fluid showed malignant cells. 

Three days later, reaccumulation of the pleural effusion occurred, necessitating a fiberoptic bronchoscopy with bronchoalveolar lavage and transbronchial biopsy of the left lower lobe lung mass. The transbronchial biopsy results were inconclusive for a diagnosis. A thyroid ultrasound was performed, showing a multinodular goiter and a 4.4-cm right lower lobe solid nodule.

The following day, she underwent a positron emission tomography (PET) scan which showed a large 18F-2-fluoro-2-deoxy-D-glucose (FDG) avid mass in the left lung base, invading the left upper and lower mainstem bronchi and a left pleural effusion. No FDG avid metastatic disease was noted. The right thyroid nodule showed no focally elevated tracer accumulation. Colonic diverticulosis was noted. We did not have access to these images, solely the reports. Thoracic surgery was consulted for an exploratory left video-assisted thoracoscopy surgery (VATS). The VATS demonstrated adhesions between the chest wall and lung, as well as multifocal areas of tumor nodules across the pleural, surface diaphragm, and mediastinum. Biopsies of the mediastinal and diaphragm tumors were taken. Sanguineous malignant pleural fluid was drained and a chest tube was placed. Serial chest x-rays were performed, and chest tube output was monitored in the following days. On postop day 2, a repeat CT of the chest/thorax showed decreased left pleural effusion, decreased mediastinal shift, and confined left lower lung lobe mass. 

Pathology results confirmed the tumor to be GIST and KIT positive. Hematology oncology was consulted. On postop day 5, she underwent a left chemical pleurodesis with doxycycline and the following day the chest tube was removed. Hematology oncology discussed treatment options with the patient. Due to the diffuse and multifocal tumor presence within the left hemithorax, surgical resection was not a treatment option. Treatment with imatinib was decided upon, and the patient was discharged with close hematology oncology follow-up.

## Discussion

GISTs are tumors originating from GI mesenchymal tissue and represent a small percentage of soft tissue tumors in the abdomen [1,6]. CD117 (c-KIT receptor) and CD34 are both important GIST biomarkers [6]. EGISTs are considered to be mesenchymal tumors originating outside the GI tract with immunohistochemical and molecular profiles similar to GIST. They account for less than 5% of GISTs [1]. GISTs manifesting in visceral organs, including liver or spleen usually, represent metastasis from a primary GI tract GIST [6]. It is rare to find a GIST arising solely in visceral organs, including the lungs. 

The first EGIST presenting above the diaphragm, with no association to the esophagus, was published in 2010. A 62-year-old male presented with a left-sided pleural-based mass unrelated to the esophagus, with extension towards the hilum. A wedge resection was performed, but three pleural-based masses recurred 10 years after, requiring resection via median sternotomy [7]. The fourth case of primary EGIST above the diaphragm was published in 2015 when a 53-year-old male who presented with severe dyspnea was found to have primary EGIST of the pericardium [8]. There was no association to the esophagus. This was the first case of EGIST originating from the pericardium. Reports of EGIST presenting in the kidney, adrenal gland, inferior vena cava, vagina, and scrotum have been found [9-13]. Diagnosis can often be delayed due to small tumor size and lack of clinical symptoms in the tumor's early stages [1,8].

The clinical presentation of pulmonary EGIST includes shortness of breath, hypoxia, and cough. CT imaging, core needle biopsy, bronchoscopy with bronchoalveolar lavage and transbronchial biopsy, and PET scan are important steps in diagnosis of pulmonary EGISTs. Diagnosis of pulmonary EGISTs primarily relies upon biopsy and immunohistochemical studies. EGIST morphology is similar to GIST with spindle cells, epithelioid cells, and polymorphic cells [1,14]. In addition, immunohistochemical stains are usually CD117 and CD34 positive; however, there are no tissue immunomarkers specific for diagnosis [1,6]. CD34 is a glycosylated transmembrane protein [1]. CD117 is a transmembrane receptor driven by c-kit and PDGFRA in the cytoplasm of the cells of Cajal [1]. Oncogenic mutations in tyrosine kinases, c-KIT or PDGFRA, are thought to cause GISTs [14]. Another explanation includes origin from ectopic interstitial cells of Cajal or pluripotent mesenchymal progenitor cells [6]. 

Treatment is with resection and tyrosine kinase inhibitors, including imatinib, regorafenib, and sunitinib [14]. Adjuvant chemotherapy with tyrosine kinase inhibitors status-post primary resection is utilized to reduce the likelihood of recurrence and metastatic GIST [1,14]. Imatinib and sunitinib suppress and block tyrosine kinase activity, preventing signal transduction [1]. 

This case presents a unique presentation of EGIST in the lung with no involvement of the GI tract. It is extremely rare for patients to be diagnosed with EGIST and even more rare for EGIST to be present above the diaphragm. This patient’s initial negative fine needle biopsy was most likely attributed to inflammation and atelectasis. Core needle biopsy showed tumor morphology and immunohistochemical staining demonstrated GIST with positive CD117, DOG-1, and CD34. CD117 is positive in 81%-100% of patients with EGIST, while CD34 is positive in 50%-70% of patients. However, DOG-1 has a positive rate of 92% in GIST patients with c-KIT mutations, suggesting a higher sensitivity and specificity. Tumor resection is usually the mainstay of therapy when possible. Imatinib can be offered when the tumor is not resectable [1]. Wedge resection and imatinib therapy in combination can be utilized [8]. 

## Conclusions

EGISTs are rare tumors with a broad range of presentations and symptoms due to their ability to affect any organ system. Pulmonary EGIST is extremely rare and diagnosis is based on biopsy and immunohistochemical staining. Therefore, proper tissue sampling is vital to diagnosis and ultimately treatment.
